# Transmission of SARS-CoV-2 from Human to Domestic Ferret

**DOI:** 10.3201/eid2709.210774

**Published:** 2021-09

**Authors:** Jožko Račnik, Ana Kočevar, Brigita Slavec, Miša Korva, Katarina Resman Rus, Samo Zakotnik, Tomaž Mark Zorec, Mario Poljak, Milan Matko, Olga Zorman Rojs, Tatjana Avšič Županc

**Affiliations:** University of Ljubljana Faculty of Veterinary Medicine, Ljubljana, Slovenia (J. Račnik, B. Slavec, O. Zorman Rojs);; Toplica Veterinary Hospital, Topolšica, Slovenia (A. Kočevar, M. Matko);; University of Ljubljana Faculty of Medicine, Ljubljana (M. Korva, K. Resman Rus, S. Zakotnik, T.M. Zorec, M. Poljak, T. Avšič Županc)

**Keywords:** COVID-19, coronavirus disease, severe acute respiratory syndrome coronavirus 2, SARS-CoV-2, viruses, pet ferret, natural infection, respiratory infections, anthropozoonosis, zoonoses

## Abstract

We report a case of natural infection with severe acute respiratory syndrome coronavirus 2 transmitted from an owner to a pet ferret in the same household in Slovenia. The ferret had onset of gastroenteritis with severe dehydration. Whole-genome sequencing of the viruses isolated from the owner and ferret revealed a 2-nt difference.

Natural infections with severe acute respiratory syndrome coronavirus 2 (SARS-CoV-2) in domestic animals living in infected households have been reported (*1*). Because of their increased popularity as a pet (*2*), domestic ferrets (*Mustela putorius furo*) pose a high risk for transmitting anthropozoonotic infections. A recent study in Spain showed that natural SARS-CoV-2 infections can occur in ferrets kept as working animals for rabbit hunting, especially if a high viral circulation is present in the human population (*3*). Further, ferrets are common laboratory animal models and, at least in experimental conditions, have been shown to be highly susceptible to SARS-CoV-2 infection and likely to transmit the virus to other ferrets without apparent clinical signs (*4*).

## The Study

On November 20, 2020, a 5-year-old neutered male domestic ferret had signs of acute gastroenteritis, including apathy, anorexia, vomiting, and profuse mucous diarrhea. Another ferret in the same household appeared healthy. Because the ferret’s condition did not improve, the owner took it to a veterinary hospital for clinical examination on November 23. The ferret was lethargic and, on the basis of skin turgor, was >5% dehydrated with low body temperature (36.4°C, reference range 37.8–40°C) and slow heart rate (180 beats/min, reference 200–400 beats/min). The body condition of the ferret was good, with a bodyweight of 1.3 kg. Several hematology and serum biochemistry results were elevated: red blood cell count (12.36 × 10^6^/µL, reference 7.01–9.65 × 10^6^/µL), hemoglobin concentration (21.2 g/dL, reference 12.2–16.5 g/dL), and hematocrit (0.57%, reference 0.36%–0.48%); blood urea nitrogen (>129.94 mg/dL, reference 18–32 mg/dL), hyperproteinemia (8.5 g/dL, reference 4.5–6.2 g/dL), hyperglobulinemia (4.4 g/dL, reference 2.8–3.6 g/dL), and borderline hyperalbuminemia (4.0 g/dL, reference 2.5–4.0 g/dL) were consistent with dehydration and possible infection. The results of all other hematologic and biochemical values were within reference ranges. Whole-body radiographs (Appendix Figure) showed splenomegaly and gas accumulation in intestinal loops. Interstitial and alveolar patterns of cranial lung lobes were present, indicating possible lobar pneumonia. The ferret was hospitalized and initially given fluid therapy, amoxicillin, esomeprazole, maropitant, and dexamethasone. Three days later, the clinical status of the ferret improved, hematologic and biochemical values normalized, and the ferret was scheduled for discharge. However, on the same day, the owner informed the veterinary hospital of having positive results for SARS-CoV-2 RNA tested on November 24, after 9 days of malaise. Additional rectal and oropharyngeal swab specimens and blood samples were taken from the ferret for further diagnostic procedures, and the ferret was discharged from the hospital and put into isolation at the owner’s home. Samples were not taken from the other pet ferret at the residence, but the rest of household members tested negative for SARS-CoV-2 RNA on November 25.

**Figure 1:**
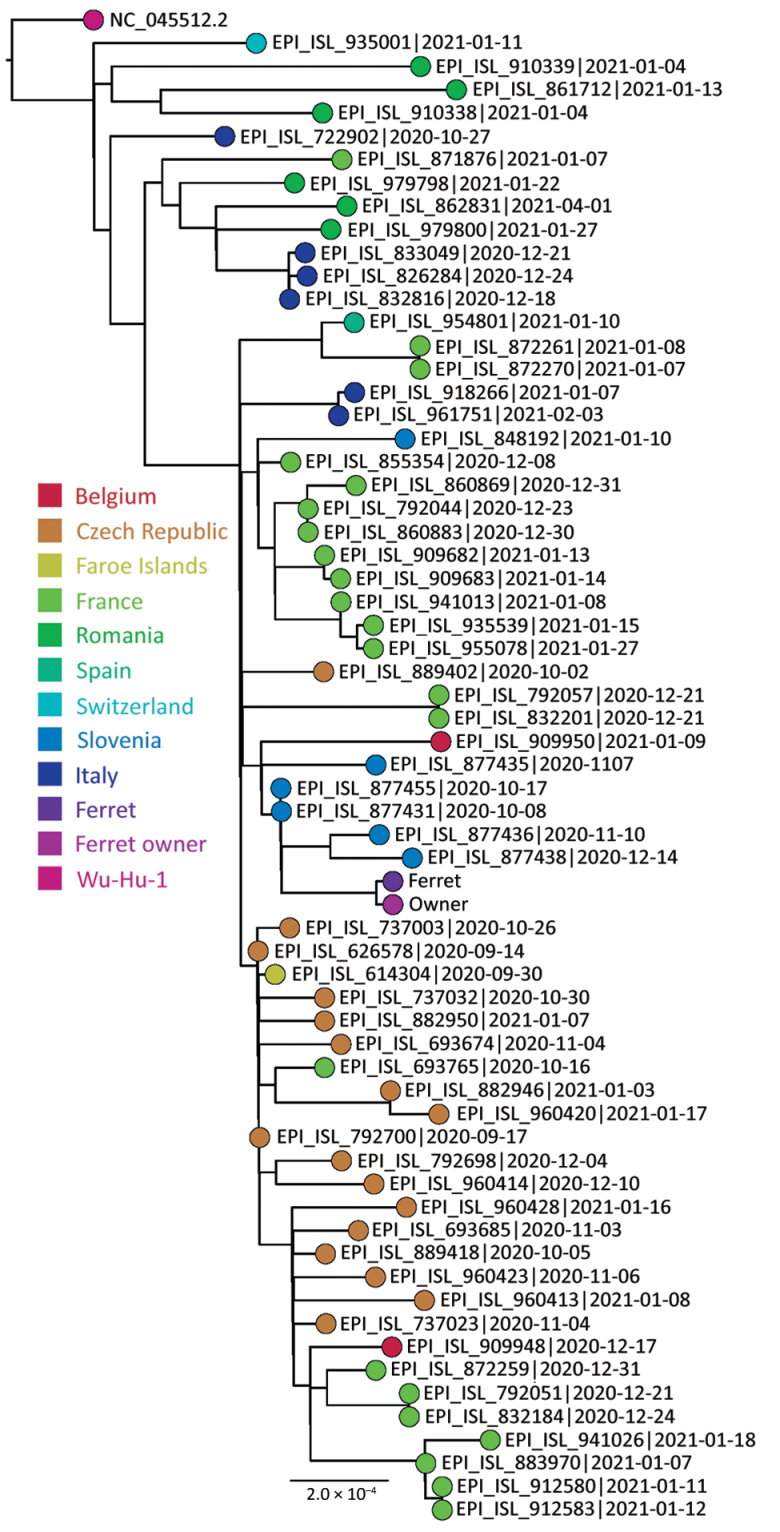
Phylogenetic sequence context consisted of high-quality complete severe acute respiratory syndrome coronavirus 2 genome sequences from a domestic ferret, Slovenia, corresponding to Pango lineage B.1.258. The context sequences were downloaded from GISAID (https://www.gisaid.org) and subsampled to 62 sequences and National Center for Biotechnology Information reference sequence NC_045512.2. The phylogenetic reconstruction using a general time-reversible plus F plus R4 substitution model was built in Figtree (Evomics, http://evomics.org) with 1,000 bootstrap replicates. The reference sequence was used as an outgroup to root the phylogenetic tree. GISAID accession numbers and isolation dates are provided. Scale bar indicates substitutions per site.

We tested the ferret’s samples for SARS-CoV-2 RNA (Appendix) and ferret-specific enteric coronavirus (FERCV) (*5*) by real-time reverse transcription PCR; influenza A and B viruses (*6*) by reverse transcription PCR; and herpesvirus (*7*), adenoviruses (*8*), and circoviruses (*9*) by PCR. The only positive result was the detection of SARS-CoV-2 RNA in the rectal and oropharyngeal swab specimens. In the oropharyngeal swab specimen, all 3 targeted genes (envelope, cycle threshold [C_t_] 27.7; RNA dependent RNA polymerase, C_t_ 28.5; and nucleocapsid, C_t_ 32.1) were detected, and in the rectal swab specimen only envelope gene (C_t_ 35.6) was detected, a finding probably attributable to a lower virus concentration. To compare the SARS-CoV-2 detected in the owner and the ferret, we conducted whole-genome sequencing on Illumina MiSeq (Illumina, https://www.illumina.com) on the basis of the ARTIC protocol (https://artic.network/ncov-2019/ncov2019-bioinformatics-sop.html). The complete genome sequences were deposited in the GISAID database (https://www.gisaid.org; accession nos. EPI_ISL_1490186 and EPI_ISL_1490187). According to the pangolin nomenclature, the sequences belonged to the B.1.258 Pango lineage, which was on the rise in Slovenia in November 2020 (Figure 1). The comparison of both sequences showed ≈100% identity, differing by 2 nucleotides (position/owner/ferret: 2,097/G/T; 22,832/C/A).

**Figure 2:**
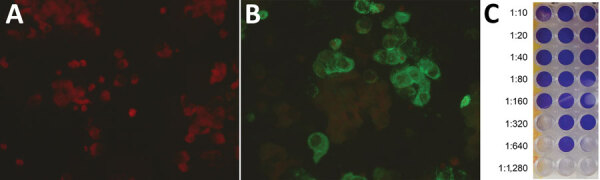
Serological response to infection with severe acute respiratory syndrome coronavirus 2 in a domestic ferret, Slovenia. Immunofluorescent tests showed a negative result in the ferret’s acute serum sample obtained on day 6 after disease onset (A) and a positive reaction at titer 1:64 in the ferret’s convalescent serum sample obtained on day 19 (B). The neutralization test (C) showed the highest dilution of the ferret’s convalescent serum sample that inhibited a cytopathic effect in >2 of 3 wells to be 1:320.

We also confirmed the SARS-CoV-2 infection in the ferret on the basis of SARS-CoV-2 IgG seroconversion and development of neutralizing antibodies. We tested the ferret’s acute and convalescent serum samples with an in-house immunofluorescent assay (Appendix). The first serum sample obtained on day 6 after disease onset tested negative; however, seroconversion was observed on day 19, when the IgG titer was 1:1,024 (Figure 2, panels A, B). In addition, we detected a high neutralizing antibody titer of 1:320 in the second serum sample (Figure 2, panel C).

## Conclusions

SARS-CoV-2 originated in animals, jumped into humans, and is now easily transmitted among humans. In addition to spreading from animals to humans, the virus can be transmitted back into animals, as observed in farmed mink (*Neovison vison*) (*10*). Most experimentally infected ferrets do not exhibit clinical signs or have only mild fever, lethargy, loss of appetite, and occasional cough (*4*,*11*). Also, among working ferrets naturally infected with SARS-CoV-2 in Spain, no signs of illness were reported (*3*).

In our study, the infected ferret had onset of severe disease with gastroenteritis, pneumonia, and dehydration and required aggressive fluid therapy and supportive care with antibiotics, antacids, antiemetics, and parenteral dexamethasone. The ferret responded to the therapy promptly and fully recovered in 3 days. Acute epizootic catarrhal enteritis caused by FERCV was one of the plausible differential diagnoses in the initial treatment plan for the ferret. For this reason, dexamethasone was used parenterally because additional treatment with a short course of steroids might speed the recovery and reduce future problems of malabsorption attributable to villi destruction caused by fulminate FERCV infection (*12*). In humans, the effective drugs against coronavirus disease are poorly defined, yet dexamethasone in combination with supportive therapy is frequently used (*13*). However, the risk for unnecessary use and adverse effects must be considered before treatment attempts with corticosteroids.

We assume that SARS-CoV-2 likely spread from the infected owner to the ferret living in the same household. Symptoms appeared in the owner 4 days before the ferret become ill. All other household members tested negative for SARS-COV-2 RNA, ruling out asymptomatic infected persons in the family. Another close contact ferret in the same household appeared healthy. Likewise, no disease among staff or animals at veterinary hospital was reported during or after the hospitalization of the ferret. Nevertheless, ferrets as laboratory models were shown to shed SARS-CoV-2 up to 8 days postinfection in nasal swab, saliva, urine, and fecal samples. Ferrets can effectively transmit the infection to other animals (*14*) or possibly humans, thus highlighting the importance of recognizing the infection in pets early to prevent spread to other animals or humans in the same household or elsewhere (*15*).

In the mink farm outbreak in Denmark, SARS-CoV-2 transmission was shown to spill over from minks to humans accumulating mutations that are resistant to neutralizing antibodies or vaccines along the way (*10*). In our study, whole-genome sequencing of the virus detected in the owner and ferret revealed only a 2-nt difference, and neither of those was present in the spike protein gene. Nonetheless, retaining the One Health approach is crucial for early detection and monitoring of emerging zoonoses in humans.

Appendix Additional information about transmission of SARS-CoV2 from human to domestic ferret.
