# Supplementary material for: Transmission of SARS-CoV-2 from Human to Domestic Ferret
Source: Emerg Infect Dis. 2021 Sep;27(9):2450–3. doi: 10.3201/eid2709.210774 (PMC8386788; doi:10.3201/eid2709.210774)
Supplement: Appendix — Additional information about transmission of SARS-CoV2 from human to domestic ferret. [file 21-0774-Techapp-s1.pdf]

Article DOI: https://doi.org/10.3201/eid2709.210774

Transmission of SARS-CoV-2 from Human to Domestic Ferret

Appendix

Materials and Methods

SARS-CoV-2 real-time reverse transcription—polymerase chain reaction (rRT-PCR)

Viral RNA was isolated with EZ1 Advanced XL using Virus Mini Kit v2.0 (Qiagen) following the manufacturer's instructions. For real-time RT-PCR amplification, we used LightMix® Modular SARS-CoV E-, RdRp-, and N-gene (Tib-Molbol, Berlin Germany) in combination with TaqMan® Fast Virus 1-Step MasterMix (Thermo Fisher Scientific, Grand Island, NY, USA) on QuantStudio 7 Pro Real-Time PCR System (Applied Biosystems, Thermo Fisher Scientific). The cycling conditions were as follows: reverse transcription at 50°C for 5 min and 95°C for 20 sec, followed by 40 cycles of PCR at 95°C for 3 sec and 60°C for 30 sec.

Whole genome sequencing

RNA was used for cDNA synthesis and PCR amplicon generation according to the nCoV-2019 sequencing protocol designed by Artic Network (https://artic.network/ncov-2019). One ng of cleaned PCR amplicon was used for synthesis of the NGS library with the Nextera XT DNA Library Preparation Kit (Illumina) following the manufacturer's instructions with an increasing number of PCR cycles in step Amplify Libraries from 12 (instructions) to 14. The NGS libraries were cleaned using a 1.8 ratio sample: Agencourt ampure XP beads (Beckman Coulter) as per instructions for short amplicons. We measured the NGS libraries' concentration with Qubit 3 (Thermo Scientific) using the Qubit dsDNA HS Assay. We measured the average fragment size and checked the NGS libraries for primer dimers with Agilent 2100 Bioanalyzer (Agilent) using the Agilent High Sensitivity DNA Kit (Agilent). NGS libraries were normalized following the manufacturer's instructions for standard normalization for the MiSeq system. In brief, all libraries were diluted to 4 nM with nuclease-free water (Qiagen), and 10 μL of each NGS library was pooled together. The pooled NGS libraries were vortexed, and 5 μL of pool was used for library denaturation and sequencing.

Immunofluorescent test

The sera were diluted from 1:16 to 1:1,024 and tested using indirect immunofluorescence assay on spot slides containing Vero E6 cells infected with SARS-CoV-2 (strain Slovenia/SI-4265/20, D614G; EVA GLOBAL Ref-SKU: 005V-03961). In brief, serum dilutions were dispensed on the antigen spot on the slide. The slide was incubated for 30 min at room temperature and washed in phosphate-buffered saline (PBS), and afterward goat anti-ferret IgG (H+L) FITC conjugate (KPL, USA) dilution at a ratio of 1:10 was added. Another incubation at room temperature followed. Then the slide was washed in PBS and dried again. The fluorescence was examination under a fluorescent microscope (Eclipse 80i; Nikon).

Neutralization test

VERO E6 cells were seeded on a 96-well plate in a concentration of 10^5 cells/mL 1 day before the neutralization test was performed. The plasma sample was incubated at 56°C for 30 min. The sample was diluted from 1:10 to 1:1,280 in DMEM with 1% FBS. SARS CoV-2 virus (strain Slovenia/SI-4265/20, D614G; EVA GLOBAL Ref-SKU: 005V-03961; 100 TCID50/mL) was added to serial dilutions in a ratio of 1:1 and incubated for 1 hour. Then 50 µL of a mixture of diluted plasma and virus was inoculated into the VERO E6 cells. The inoculated cells were incubated for 4 days at 37°C and 5% CO_2. On day 5, the cells were observed for CPE and the antibody titer was detected as the highest dilution that CPE was inhibited on for at least 2 of 3 wells.

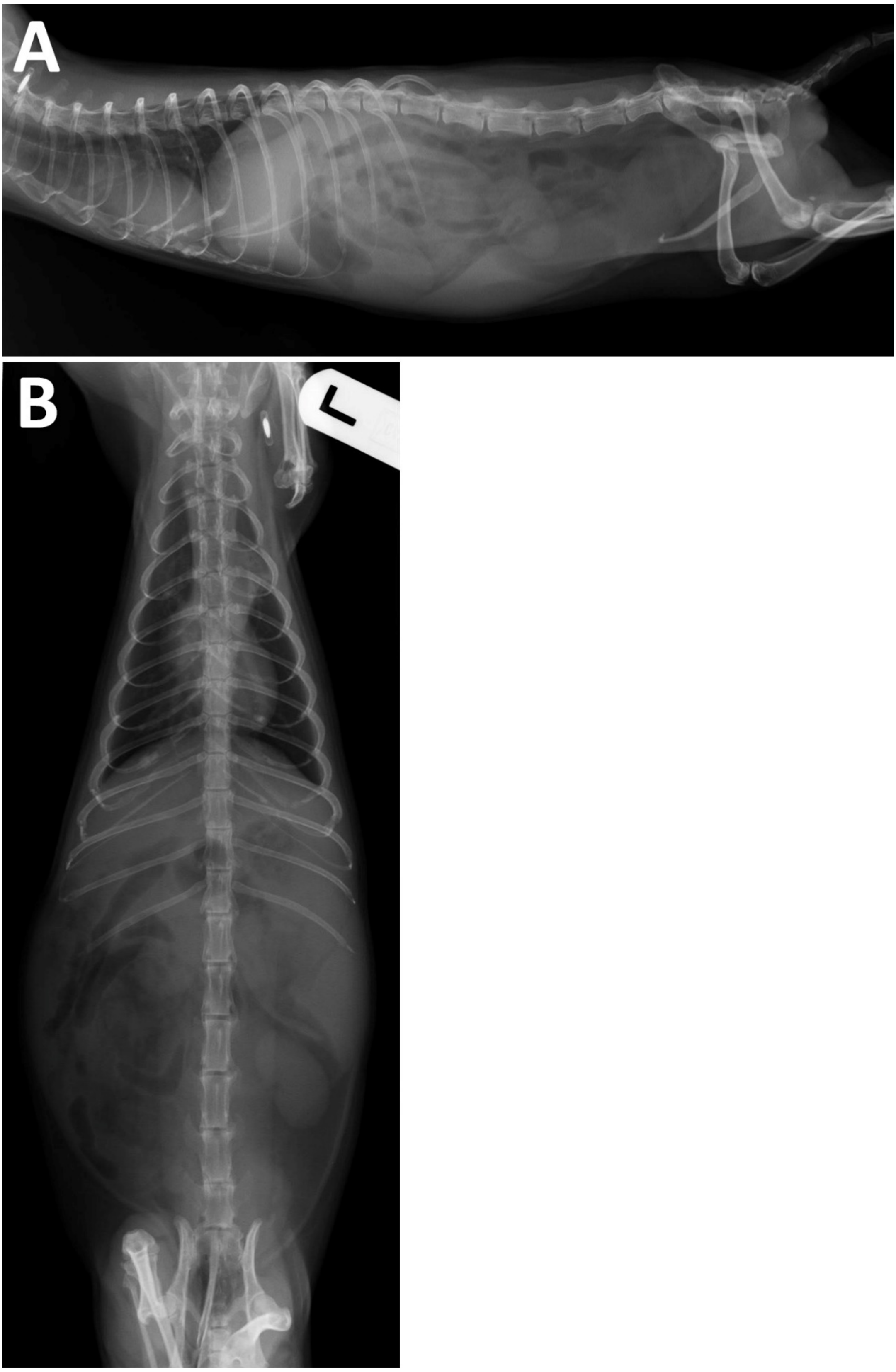

Appendix Figure. Whole-body radiograph in laterolateral (A) and ventrodorsal (B) projections of a ferret presenting splenomegaly and gas accumulation in intestinal loops. Moreover, interstitial and alveolar patterns of cranial lung lobes are present, indicating possible lobar pneumonia.
